# Evidence against solar influence on nuclear decay constants

**DOI:** 10.1016/j.physletb.2016.08.038

**Published:** 2016-08-24

**Authors:** S. Pommé, H. Stroh, J. Paepen, R. Van Ammel, M. Marouli, T. Altzitzoglou, M. Hult, K. Kossert, O. Nähle, H. Schrader, F. Juget, C. Bailat, Y. Nedjadi, F. Bochud, T. Buchillier, C. Michotte, S. Courte, M.W. van Rooy, M.J. van Staden, J. Lubbe, B.R.S. Simpson, A. Fazio, P. De Felice, T.W. Jackson, W.M. Van Wyngaardt, M.I. Reinhard, J. Golya, S. Bourke, T. Roy, R. Galea, J.D. Keightley, K.M. Ferreira, S.M. Collins, A. Ceccatelli, M. Unterweger, R. Fitzgerald, D.E. Bergeron, L. Pibida, L. Verheyen, M. Bruggeman, B. Vodenik, M. Korun, V. Chisté, M.-N. Amiot

**Affiliations:** a European Commission, Joint Research Centre (JRC), Retieseweg 111, B-2440Geel, Belgium; b Physikalisch-Technische Bundesanstalt (PTB), Bundesallee 100, 38116 Braunschweig, Germany; c Institut de Radiophysique, Lausanne (IRA), Switzerland; d Bureau International des Poids et Mesures (BIPM), Pavillon de Breteuil, 92310 Sèvres, France; e Radioactivity Standards Laboratory (NMISA), 15 Lower Hope Road, Rosebank 7700, Cape Town, South Africa; f National Institute of Ionizing Radiation Metrology (ENEA), Casaccia Research Centre, Via Anguillarese, 301—S.M. Galeria I-00060 Roma, C.P. 2400, I-00100 Roma A.D., Italy; g Australian Nuclear Science and Technology Organisation (ANSTO), Locked Bag 2001, Kirrawee, NSW 2232, Australia; h National Research Council of Canada (NRC), 1200 Montreal Road, Ottawa, ON, K1A0R6, Canada; i National Physical Laboratory (NPL), Hampton Road, Teddington, Middlesex TW11 OLW, UK; j Terrestrial Environment Laboratory, IAEA Environment Laboratories, Department of Nuclear Sciences and Applications, International Atomic Energy Agency (IAEA), Vienna International Centre, PO Box 100, 1400 Vienna, Austria; k Physical Measurement Laboratory, National Institute of Standards and Technology (NIST), 100 Bureau Dr., Gaithersburg, MD 20899-8462, USA; l Belgian Nuclear Research Centre (SCK·CEN), Boeretang 200, B-2400 Mol, Belgium; m Jožef Stefan Institute (JSI), Jamova 39, 1000 Ljubljana, Slovenia; n CEA, LIST, Laboratoire National Henri Becquerel (LNHB), Bât. 602 PC 111, CEA-Saclay 91191 Gif-sur-Yvette cedex, France

**Keywords:** Half-life, Decay constant, Uncertainty, Radioactivity, Sun, Neutrino

## Abstract

The hypothesis that proximity to the Sun causes variation of decay constants at permille level has been tested and disproved. Repeated activity measurements of mono-radionuclide sources were performed over periods from 200 days up to four decades at 14 laboratories across the globe. Residuals from the exponential nuclear decay curves were inspected for annual oscillations. Systematic deviations from a purely exponential decay curve differ from one data set to another and are attributable to instabilities in the instrumentation and measurement conditions. The most stable activity measurements of alpha, beta-minus, electron capture, and beta-plus decaying sources set an upper limit of 0.0006% to 0.008% to the amplitude of annual oscillations in the decay rate. Oscillations in phase with Earth’s orbital distance to the Sun could not be observed within a 10^−6^ to 10^−5^ range of precision. There are also no apparent modulations over periods of weeks or months. Consequently, there is no indication of a natural impediment against sub-permille accuracy in half-life determinations, renormalisation of activity to a distant reference date, application of nuclear dating for archaeology, geo- and cosmochronology, nor in establishing the SI unit becquerel and seeking international equivalence of activity standards.

## 1. Introduction

The exponential-decay law is one of the most famous laws of physics, already carved in stone since the pioneering work of Ernest Rutherford [1], Maria Skłodowska-Curie [2] and others. It has withstood numerous tests [3–5] demonstrating that the decay of a radionuclide can be characterised solely by a single decay constant – or equivalently by the half-life – which is invariable in space and time. However, observations of periodic oscillations in measured decay rates of radioactive sources [6–13] have been heavily debated in the last decade [6–25]. Controversy arose at two levels: (i) at the observational level, with experimental data sets showing significant differences in stability of decay rates with time, and (ii) at the interpretational level, either ascribing the observed modulations to instabilities in the detection system, or advocating new physics to explain variability in the decay constants.

As much as the instability claims attract interest as inspiration for new physical theories and applications [14,15], if true they would have major implications on traceability and equivalence in the common measurement system of radioactive substances. Variability of decay constants at permille level would limit the precision by which a half-life value could be assigned to a radionuclide, as well as the accuracy by which the SI-unit becquerel could be established through primary standardisation [26] and international equivalence demonstrated through key comparisons and the Système International de Référence (SIR) [27]. The implications at metrological level would eventually affect science built on the decay laws, from renormalisation of activity to a reference date for nuclear dosimetry to precise nuclear dating for geo- and cosmochronology.

At the heart of this controversy are the metrological difficulties inherent to the measurement of half-lives [28–30]. From a metrological point of view, it is obvious that instruments, electronics, geometry and background may vary due to external influences such as temperature, pressure, humidity and natural or man-made sources of radioactivity. Claims of variability of half-lives on the basis of deviations from an exponential decay curve can only be considered when the instrumental effects have been fully compensated and/or accounted for in the uncertainty budget. Jenkins et al. [9] claim to have done so before proposing their hypothesis that permille sized seasonal variations of decay rates of ^226^Ra and ^36^Cl are caused by solar influences on their decay constants [6–8]. Evidence has been collected to demonstrate instabilities in the decay of other radionuclides [10,11] and by means of time-frequency analysis periodicity at shorter and longer term than 1 year have been claimed [11–13]. However, this interpretation is being challenged by the publication of data sets confirming a close adherence to exponential decay with residuals in the 10^−5^ range [16,18,20,21,23].

Authors of both convictions expressed the need for collecting evidence for different radionuclides measured with different detection techniques [7,11,13,18,23]. At national metrology institutes (NMIs) taking responsibility for establishing the unit becquerel, mono-radionuclide sources are kept and regularly measured for standardisation purposes as well as for determining half-lives. In addition, gamma-ray spectrometry laboratories keep records of quality control measurements on their spectrometers which provide useful information on long-term trends in activity measurements of a reference source. In this work, the hypothesis that decay constants vary through solar influence in phase with Earth–Sun orbital distance has been tested through the analysis of a unique collection of activity measurements repeated over periods of 200 days up to four decades at 14 laboratories distributed across the globe.

## 2. Measurements & analysis

Precise activity measurement series were performed for alpha decay (^209^Po, ^226^Ra series, ^228^Th, ^230^U, ^241^Am), beta minus decay (^3^H, ^14^C, ^60^Co, ^85^Kr, ^90^Sr, ^124^Sb, ^134^Cs, ^137^Cs), electron capture (^54^Mn, ^55^Fe, ^57^Co, ^82,85^Sr, ^109^Cd, ^133^Ba), a mixture of electron capture and positron decay (^22^Na, ^65^Zn, ^207^Bi), and a mixture of electron capture and beta minus/plus decay (^152^Eu). More than 60 data sets were collected, some of which were performed over several decades. Some data sets excel in precision, others reveal vulnerability of different measurement techniques to external conditions. Characteristics of the data sets are summarised in Table 1.

The measurement techniques employed are as follows: ionisation current measurements in a re-entrant ionisation chamber (IC) or a hospital calibrator (HIC) [31,32], net area analysis of full-energy γ-ray peaks (and integral spectrum counting) by γ-ray spectrometry with a HPGe detector (HPGe) [33], particle counting in a planar silicon detector in quasi-2πconfiguration (PIPS) [34], X-ray counting at a small defined solid angle with a gas-filled proportional counter (PC) [35,36], live-timed β–γ anti-coincidence counting (LTAC) [37], triple-to-double coincidence counting with a liquid scintillation vial and three photodetectors (TDCR) [38], liquid scintillation counting (LSC) [38], particle and photon counting in a sandwich CsI (Tl) spectrometer (CsI) [39], internal gas counting (IGC) [40], and α-particle counting at a small defined solid angle with a large planar silicon detector (αDSA) [35,36]. An overview of standardisation techniques and their sources of error can be found in the special issues 44(4) and 52(3) of Metrologia [41,42] and references in [25,28].

Exponential decay curves were fitted to the data and the residuals were inspected for annual modulations. The data sets were first compensated for (1) the presence of occasional outlier values, (2) abrupt systematic changes in the detector response, e.g. due to replacement of the electronics or recalibrations of the instrument, and (3) systematic drift extending over periods of more than 1 year, e.g. due to gas loss from an ionisation chamber, uncompensated count loss through pulse pileup in a spectrometer, activity build-up from decay products in a source, etc. The residuals were binned into 8-day periods of the year and averaged to obtain a reduced set of (maximum) 46 residuals evenly distributed over the calendar year. To the averaged residuals, a sinusoidal shape *A* sin(2π(*t*+*a*)/365) has been fitted in which *A* is the amplitude, *t* is the elapsed number of days since New Year, and *a* is the phase shift expressed in days. The fitted amplitude values can be considered insignificant if they are of comparable magnitude as their estimated standard uncertainty (see Table 1).

## 3. Discussion

**Fig. 1:**
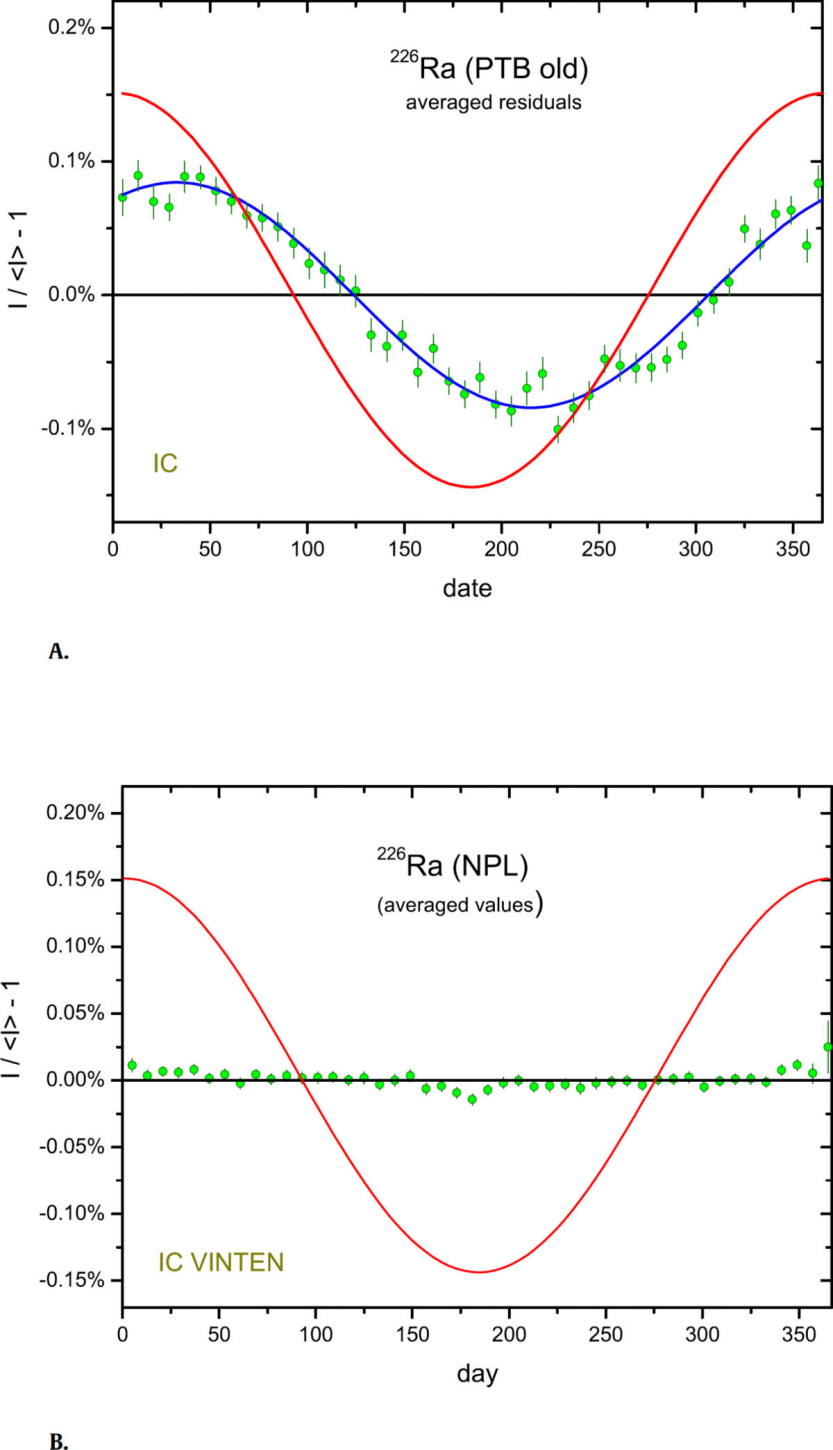
**A.** Annual average residuals from exponential decay for ^226^Ra activity measurements with an IC at PTB from 1983 to 1998. The line represents relative changes in the inverse square 1/*R*^2^ of the Earth–Sun distance, normalised to an amplitude of 0.15%. **B.** Same for ^226^Ra activity measurements with the Vinten IC of NPL from 1993 to 2016, after renormalisation per calendar year.

**Fig. 2:**
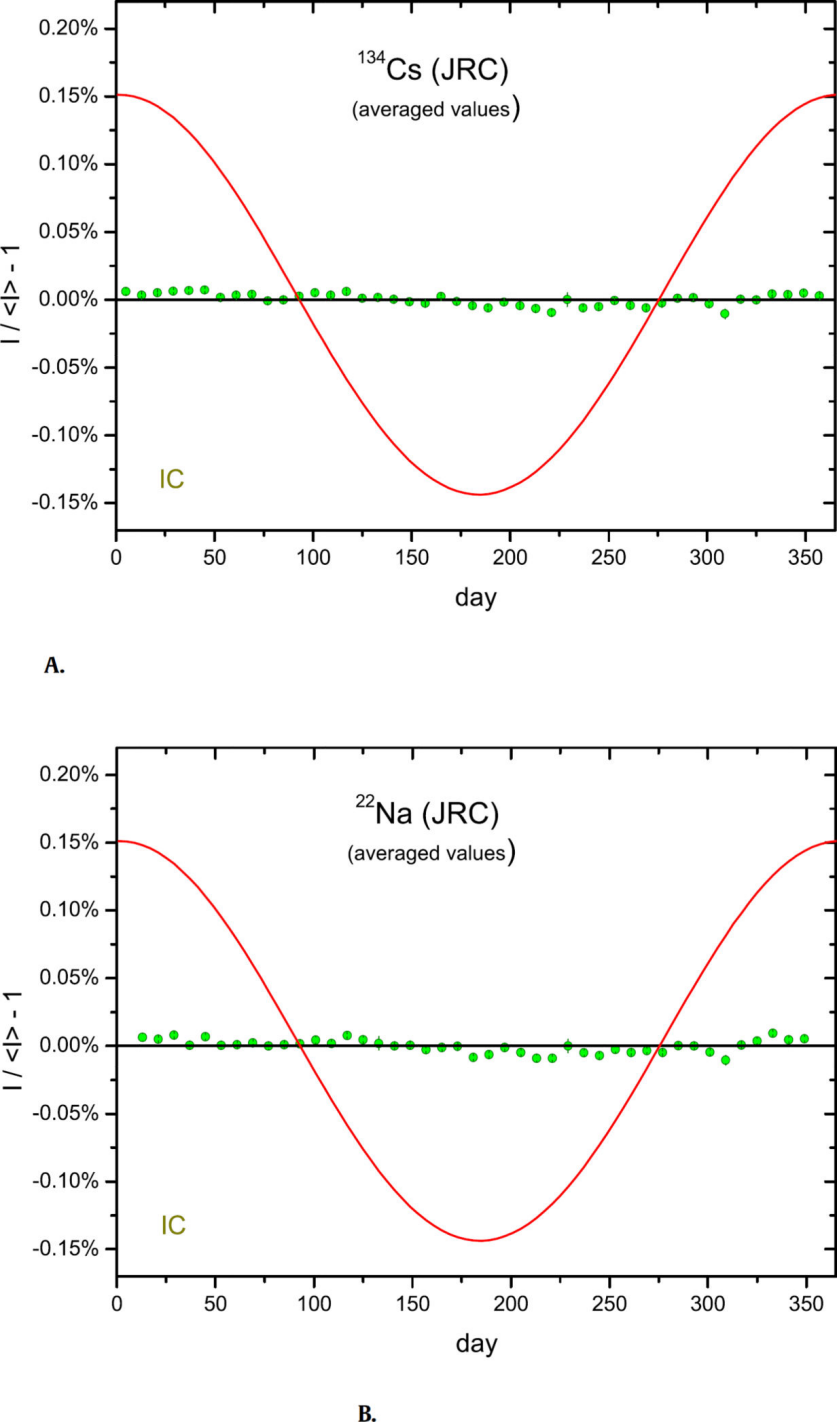
**A.** Annual average residuals from exponential decay for ^134^Cs activity measurements with the IG12 IC at the JRC from 2010 to 2016. **B.** Same for ^22^Na.

The controversy started with the interpretation [7,8] of *A* ≈ 0.15% modulations in the decay rate measurements of a sealed ^226^Ra reference source in an IC at the PTB between 1983 and 1998. The averaged residuals, shown in Fig. 1A, have a sinusoidal shape with amplitude *A* = 0.083 (2)% and phase *a* = 59 days. An explanation through solar influence on the alpha or beta decay constants of nuclides in the ^226^Ra decay series seems unlikely, since the residuals are out of phase with the annual variation of the inverse square of the Sun–Earth distance, 1/*R*^2^ (renormalised to 0.15% amplitude in the Figs. 1–2 of this work). The real cause is of instrumental nature, since the modulations were significantly reduced after changing the electrometer of the IC [22,25]. There is a remarkable correlation with average seasonal changes of radon concentration in air (*A* = 16 (2)%, *a* = 57 days) measured inside the laboratory from 2010 to 2016, but causality has not been proven.

At other institutes, annual modulations of smaller amplitude and different phase have been observed, which demonstrates the local character of the non-exponential behaviour. The data sets for ^226^Ra show a different level of instrumental instability, but the most stable ^226^Ra measurements prove invariability of its decay constant against annual modulations within 0.0025% to 0.005%. An example is shown in Fig. 1B, comprising 4000 ^226^Ra ionisation current measurements over a period of 22 years at the NPL.

Stability is best achieved where the detector efficiency is least influenced by geometrical and environmental variations and where the signal of the radiation is easily separated from interfering signals and electronic noise. For example, measuring ^241^Am decay through alpha-particle detection with close to 100% detection efficiency would typically be more stable than through fractional detection of its low-energy photon emissions in a gas-filled pro-portional counter. For the alpha emitters, ^209^Po, ^226^Ra, ^230^U, and ^241^Am, the invariability of the decay constants was confirmed within the 10^−5^ level.

Comparably lower stability could be anticipated for beta-minus decay. Parkhomov [10] found 7 data sets of beta-decaying radionuclides exhibiting periodic variations of 0.1% to 0.3% amplitude with a period of 1 year. Fischbach et al. [8,14,15] suggested new theories in which the variable flux of anti-neutrinos from the Sun would significantly modulate the probability for β^−^ emission. From metrological point of view, instability in the detection efficiency for a pure beta emitter can be expected due to the continuous energy distribution of the beta particle which makes the count rate subject to threshold variations at the low-energy side and possibly incomplete detection probability at the high-energy side. However, measurements based on γ-ray emission subsequent to the β^−^ emission – possibly through the decay of a short-lived daughter nuclide – can be made more robust.

High-quality measurement data were collected for β^−^ emitters in Table 1, mostly obtained by IC but also with primary activity measurement techniques such as the triple-to-double coincidence ratio (TDCR) method and live-timed 4π β–γ anti-coincidence counting (LTAC). It was demonstrated for ^36^Cl [20], ^60^Co (Table 1) and ^90^Sr/^90^Y [23] that primary standardisation techniques like TDCR and LTAC are more stable than routine counting techniques, because each measurement provides information about the detection efficiency and automatically corrects for its fluctuations. Some IC measurements show remarkable stability, too, and refute the conclusions made about variability of the decay constants as well as the hypothesis of a significant solar influence on the decay rate. In Fig. 2A, averaged residuals for ^134^Cs in an IC demonstrate stability within the 10^−5^ range. Evidence of stability down to the 10^−5^ level was found for the beta minus emitters ^60^Co, ^90^Sr, ^124^Sb, ^134^Cs and ^137^Cs, and down to the 10^−4^ level for ^3^H, ^14^C, and ^85^Kr. These results are in direct contradiction with the permille level oscillations for ^3^H, ^60^Co, ^90^Sr, and ^137^Cs reported by Parkhomov [10] and Jenkins et al. [11].

**Fig. 3:**
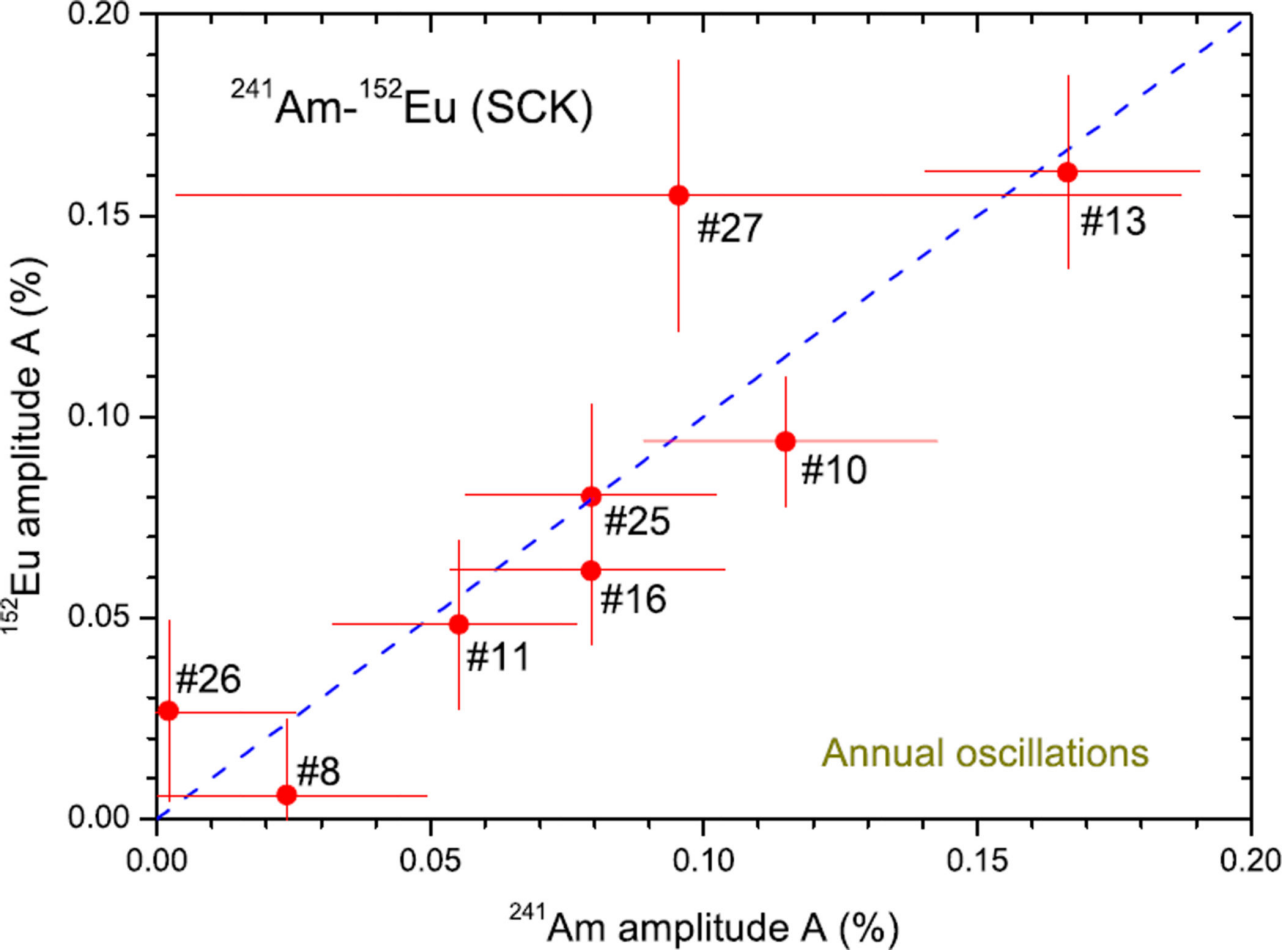
Amplitude of average annual oscillations in the decay rates of ^241^Am and ^152^Eu measured by γ-ray spectrometry with 8 HPGe detectors at SCK between 2008 and 2016. The index refers to the detector number. A mixed ^241^Am– ^152^Eu point source was measured 166–466 times in a fixed geometry at about 11 cm from the endcap using the 59 keV line of ^241^Am and the 122 keV, 779 keV and 1408 keV lines of ^152^Eu.

Radionuclides disintegrating by electron capture (EC) and β^+^ decay – ^22^Na, ^54^Mn, ^55^Fe, ^57^Co, ^65^Zn, ^82^Sr/^82^Rb+^85^Sr, ^109^Cd, ^133^Ba, ^152^Eu, and ^207^Bi – were investigated by the same techniques as α and β^−^ emission and, also here, stability within the 10^−5^ to 10^−4^ range was observed in most cases. An example is shown in Fig. 2B for ^22^Na measured in the same period with the same IC as ^134^Cs in Fig. 2A. The tiny modulations in the residuals for both nuclides are highly correlated, which is most likely a seasonal effect of instrumental origin. Clear evidence of annual modulations being of instrumental origin has been found in thousands of γ-ray spectrometry measurements with 8 HPGe detectors at the SCK, as shown in Fig. 3: the modulations in measured decay rates for the alpha decay of ^241^Am and mixed EC, β^−^, and β^+^ decay of ^152^Eu are highly correlated but the amplitude differs from one detector to another. In other words, the modulations are linked to the instrument, not to the type of decay.

## 4. Conclusions

The experimental data in this work are typically 50 times more stable than the measurements on which recent claims for solar influence on the decay constants were based. The observed seasonal modulations can be ascribed to instrumental instability, since they vary from one instrument to another and show no communality in amplitude or phase among – or even within – the laboratories. The exponential decay law is immune to changes in Earth–Sun distance within 0.008% for most of the investigated α, β^−^, β^+^ and EC decaying nuclides alike.

Owing to the invariability of decay constants, there is no impediment to the establishment of the becquerel through primary standardisation at 0.1% range accuracy nor to the demonstration of equivalence of activity at international level over a time span of decades. It is normal for repeated activity measurements to show varying degrees of instability of instrumental and environ-mental origin and such auto-correlated variability should be taken into account next to statistical variations when setting alarm levels in quality control charts. Taking into account such instabilities and adhering to proper uncertainty propagation, no fundamental objections need to be made against half-life measurement with sub-permille uncertainties, nor against applying exponential decay formulas to calculate activity at a future or past reference time or to perform accurate nuclear dating.

## Figures and Tables

**Table1:** Characteristics of the decay rate measurement sets analysed. The method acronyms are explained in the text. The period indicates the first and last year in which data were collected. The standard deviation is an indication of the uncertainty on the annual averaged data (maximum 46 data, covering 8-day periods), derived from the spread of the input data and the inverse square root of the number of values in each data group. The amplitude and phase are the result of the fit of a sinusoidal function to the averaged data. In bold are the amplitudes at 10^−6^–10^−5^ level. The estimated standard uncertainty on the amplitude is indicated between parentheses, its order of magnitude corresponding to that of the last digit of the value of *A*.

Decay mode(s)	Nuclide	Laboratory	Method	Period (year)	#Data	Rel. std dev in %	Amplitude *A* in %	Phase shift α in days
α	^209^Po	JRC	PIPS	2013–2016	1539	0.024	**0.006** (5)	6
α + β^−^	^226^Ra	PTB	IC	1983–1998	1973	0.011	0.083 (2)	59
α + β^−^	^226^Ra	PTB	IC	1999–2016	2184	0.005	0.016 (1)	194
α + β^−^	^226^Ra	ENEA	IC	1992–2015	161	0.025	0.043 (5)	324
α + β^−^	^226^Ra	NIST	IC #1	2008–2016	99	0.016	0.015 (3)	255
α + β^−^	^226^Ra	NIST	IC #2	2012–2016	272	0.036	**0.002** (8)	8
α + β^−^	^226^Ra	BIPM	IC	2001–2015	136	0.015	**0.004** (3)	4
α + β^−^	^226^Ra	JRC	IC	2005–2015	1737	0.005	**0.003** (2)	363
α + β^−^	^226^Ra	NPL	IC #1	1993–2016	4055	0.014	**0.0025** (18)	60
α + β^−^	^226^Ra	NPL	IC #2	1993–2016	3996	0.005	**0.005** (1)	73
α + β^−^	^226^Ra	NMISA	IC	1992–2015	276	0.343	0.106 (60)	67
α + β^−^	^226^Ra	ANSTO	IC	2012–2015	700	0.015	**0.005** (3)	256
α + β^−^	^226^Ra	ANSTO	HIC	2008–2014	1749	0.077	0.009 (18)	82
α + β^−^	^226^Ra	LNHB	IC #1	1998–2016	455	0.026	0.026 (6)	328
α + β^−^	^226^Ra	LNHB	IC #2	1998–2016	498	0.028	0.042 (7)	294
α	^228^Th	NIST	IC	1968–1978	70	0.107	0.031 (22)	327
α	^230^U	JRC	αDSA, PIPS, CsI, LSC, HPGe	2010–2011	5451	0.083	**0.007** (7)	173
α	^241^Am	JRC	PC	2004–2008	245	0.022	0.101 (16)	104
α	^241^Am	SCK	HPGe #8	2008–2016	430	0.13	0.024 (28)	55
α	^241^Am	SCK	HPGe #26	2013–2016	166	0.12	**0.002** (23)	304
α	^241^Am	SCK	HPGe #11	2008–2016	402	0.12	0.055 (22)	242
α	^241^Am	SCK	HPGe #16	2008–2016	382	0.13	0.079 (26)	290
α	^241^Am	SCK	HPGe #25	2011–2016	245	0.12	0.079 (22)	236
α	^241^Am	SCK	HPGe #10	2008–2016	466	0.14	0.115 (27)	259
α	^241^Am	SCK	HPGe #27	2011–2015	238	0.45	0.095 (91)	280
α	^241^Am	SCK	HPGe #13	2008–2016	434	0.12	0.167 (26)	235
α	^241^Am	PTB	LSC	2014–2016	574	0.004	**0.0006** (7)	260
β^−^	^3^H	JRC	LSC	2002–2014	706	0.112	0.048 (24)	197
β^−^	^3^H	NIST	IGC	1961–1999	21	0.75	0.18 (20)	149
β^−^	^14^C	JRC	LSC	2002–2014	706	0.075	0.013 (16)	92
β^−^	^14^C	NMISA	TDCR	1994–2014	32	0.250	0.067 (80)	59
β^−^	^60^Co	NIST	IC	1968–2007	250	0.050	**0.007** (7)	0
β^−^	^60^Co	NIST	LTAC + IC	2006–2014	26 + 7	0.036	**0.007** (9)	18
β^−^	^60^Co	JSI	HPGe #1–6	1998–2016	15254	0.079	0.041 (14)	161
β^−^	^85^Kr	NIST	IC	1980–2007	98	0.035	0.036 (15)	153
β^−^	^90^Sr	PTB	TDCR	2013–2014	4493	0.009	**0.004** (2)	362
β^−^	^90^Sr	PTB	IC	1989–2016	2207	0.009	0.018 (2)	26
β^−^	^124^Sb	JRC	IC	2007	59	0.005	**0.003** (2)	241
β^−^	^134^Cs	JRC	IC	2010–2015	1065	0.002	**0.0051** (5)	48
β^−^	^137^Cs	IRA	IC	1984–2012	276	0.043	0.018 (9)	342
β^−^	^137^Cs	NRC	IC #1–3	1995–2009	62	0.074	**0.006** (22)	147
β^−^	^137^Cs	PTB	IC	1997–2016	2149	0.005	0.014 (1)	29
β^−^	^137^Cs	NIST	IC	1968–2011	254	0.034	**0.004** (6)	33
β^+^, EC	^22^Na	JRC	IC	2010–2016	443	0.003	**0.0047** (6)	53
EC	^54^Mn	JRC	IC	2006–2009	156	0.007	**0.005** (1)	28
EC	^54^Mn	PTB	IC	2010–2016	716	0.011	0.014 (2)	78
EC	^55^Fe	JRC	IC	2004–2005	595	0.007	**0.004** (3)	187
EC	^57^Co	NIST	IC	1962–1966	97	0.089	0.055 (22)	324
EC, β^+^	^65^Zn	JRC	IC	2002–2003	140	0.026	**0.008** (4)	163
EC(, β^+^)	^82^Sr/^82^Rb + ^85^Sr	NIST	IC	2007–2008	158	0.011	**0.0006** (27)	240
EC(, β^+^)	^82^Sr/^82^Rb	NIST	HPGe	2007–2008	23	0.46	0.073 (75)	255
EC	^109^Cd	JRC	IC	2006–2010	125	0.017	0.015 (4)	18
EC	^109^Cd	JSI	HPGe #3, 4	1998–2016	5414	0.139	0.035 (24)	346
EC	^109^Cd	NIST	IC	1976–1981	167	0.058	0.013 (15)	220
EC	^133^Ba	NIST	IC	1979–2012	131	0.042	0.028 (8)	74
EC, β^−^, β^+^	^152^Eu	IAEA	HPGe #1, 2	2010–2016	143	0.113	0.020 (24)	162
EC, β^−^, β^+^	^152^Eu	SCK	HPGe #8	2008–2016	1228	0.10	**0.006** (19)	242
EC, β^−^, β^+^	^152^Eu	SCK	HPGe #26	2013–2016	499	0.10	0.027 (23)	178
EC, β^−^, β^+^	^152^Eu	SCK	HPGe #11	2008–2016	1168	0.10	0.048 (21)	280
EC, β^−^, β^+^	^152^Eu	SCK	HPGe #16	2008–2016	1260	0.08	0.062 (18)	285
EC, β^−^, β^+^	^152^Eu	SCK	HPGe #25	2011–2016	723	0.10	0.080 (23)	213
EC, β^−^, β^+^	^152^Eu	SCK	HPGe #10	2008–2016	1374	0.08	0.094 (16)	206
EC, β^−^, β^+^	^152^Eu	SCK	HPGe #27	2011–2015	698	0.16	0.155 (34)	228
EC, β^−^, β^+^	^152^Eu	SCK	HPGe #13	2008–2016	1249	0.11	0.161 (24)	214
EC, β^−^, β^+^	^152^Eu	NIST	IC	1976–2011	96	0.040	0.021 (9)	214
EC, β^−^, β^+^	^152^Eu	PTB	IC	1989–2016	2199	0.007	0.018 (1)	11
EC(, β^+^)	^207^Bi	NIST	IC	1971–2011	152	0.05	**0.004** (11)	23
